# Dental Prosthetic Rehabilitation Interventions in Elderly Patients Hospitalized in the Nursing Homes of the Lombardy Region: A Retrospective Study

**DOI:** 10.3390/healthcare10112328

**Published:** 2022-11-21

**Authors:** Saverio Ceraulo, Paolo Caccianiga, Carmelo Casto, Ignazio Ceraulo, Gianluigi Caccianiga

**Affiliations:** 1 School of Medicine and Surgery, University of Milano-Bicocca, 20900 Monza, Italy; 2 Independent Researcher, 73000 Lecce, Italy; 3 Independent Researcher, 20100 Milan, Italy

**Keywords:** prosthodontics, nursing homes, teledentistry, fragile patients, dental rehabilitation

## Abstract

*Background*: The difficulty of frail, non-self-sufficient or non-ambulatory collaborating elderly residents in nursing homes to eat due to a lack of teeth or the absence of a dental prosthesis leads to an increasingly evident increase in the patients’ systemic pathological state, particularly in older patients who take a lot of medications. Total or partial edentulousness that is not filled with dental prostheses or that is filled with inadequate prostheses, associated with socioeconomic factors, depression, impaired motor functions, heart disease and a large number of chronic diseases including excessive use of drugs, often affects elderlies’ feeding. *Aim*: In this study, prosthetic rehabilitation was performed on some frail elderly residents in 10 nursing homes in the Lombardy district, and, subsequently, meal behavior and social activity were examined in two information questionnaires through compilation. *Methods*: The research was conducted on only 67 patients, 26 men and 41 women, aged between 75 and 99, who were guests in 10 health facilities (nursing homes) in the Lombard district; only 8 residents did not undergo prosthetic rehabilitation, as they did not cooperate. All the patients who were visited underwent oral prosthetic rehabilitation, and, subsequently, some aspects such as nutrition and socialization were assessed with other residents through the aid of two information dossiers. *Results*: the results showed that all the residents, despite difficulties in chewing with the new prosthesis, were fed and did not refuse more consistent foods; in addition, there was an improvement in social activity among the residents. Only 19.3% of men and 22% of women continued to eat little; there was an improvement in the participation in social activities among the residents, with a percentage of 73% of men and 88% of women; in particular, during meals 35 residents conversed with the other residents. *Discussion*: The dental problems of elderly people residing in social welfare homes are increasingly evident when other systemic pathologies are present. It would be desirable to introduce telemedicine in residences for the elderly for the monitoring of dental problems. *Conclusions*: From the information obtained and from the evaluation of the change in the elderly, it can be concluded that it would be desirable to include specific dental protocols to create a network, including a telematic one, to monitor and perform more dental checks in nursing homes.

## 1. Introduction

Nutrition is the prerequisite for the construction and function of the organism, and its importance must also be considered from the point of view of preventive medicine. There is a connection between the frailty of the elderly, sarcopenia and nutrition. The life of a body is fueled by nourishment. An alteration in the manner in which one eats inevitably translates into an alteration of body fluids, muscle mass, and, more deeply, the energy stored and available for all bodily activities. The elderly population is rapidly increasing around the world. Proper nutrition allows each subject to maintain health and well-being. There are many factors that influence the nutritional status of the elderly. Factors such as physiological changes that occur with aging, socioeconomic factors, dementia, depression, impaired motor function, a large number of chronic diseases, overuse of drugs and low food intake due to disease affect the nutrition of the elderly [1]. Cardiovascular diseases such as hypertension, ischemic heart disease and heart failure are more common in the elderly, and nutrition is important in relation to these diseases in terms of mortality and morbidity [2]. In some populations, significant geographic differences within individual countries are noted if polydrug intake is considered, and this supports the continuing need for a worldwide comprehensive geriatric assessment [3]. Chewing and insalivation are important steps in the processes of digestion and assimilation of food; the variety of dental problems faced by the elderly population can lead to difficulties in these fundamental processes, leading to changes in the selection of foods to be consumed, itself leading to malnutrition and sarcopenia [4]. Poor oral hygiene and poor oral health, moreover, can predispose to chronic inflammation of the lower airways through periodontitis [5], which is a recognized pathophysiological factor in the onset of sarcopenia. In recent years, it has been highlighted that sarcopenia is a phenomenon that affects not only the muscles of the lower limbs, but those of the whole body, including the muscles involved in chewing and swallowing, with a negative impact on food intake [6,7]. The Corona Virus pandemic (COVID-19) has changed many habits in populations, and in some people, the different waves have highlighted the fear of being infected even more [8,9,10,11]. The continuous increase in the elderly population and the greater availability of new techniques have increased both requests for dental therapy and the quality of the services required. From this perspective, the implant-prosthetic rehabilitation of geriatric patients is becoming more and more frequent, as is the maintenance of optimal conditions of the oral cavity through caring for the periodontal tissue [12,13,14], thus guaranteeing a better quality of life. Although the new implant surgical techniques allow one to even carry out rehabilitations in unfavorable conditions, i.e., maxillary sinus lifts and bone grafts, it is not always possible to subject geriatric patients to long and complex sessions that could be too demanding for their physical state of fragility or psychophysical balance. In the geriatric panorama, the figure of the dentist becomes very important for all those frail people who show signs of psychophysical decay, in particular for the guests of nursing homes with non-hospital structures but with, in any case, a health imprint, which for a variable period ranging from a few weeks to an indefinite period host non-self-sufficient people who cannot be cared for at home and who need specific medical care from several specialists and comprehensive healthcare. Proper nutritional therapy in combination with exercise, optimal glycemic and metabolic control, and social participation/support for the prevention of frailty can extend a healthy life expectancy and maintain a quality of life in elderly people with metabolic disorders [15,16,17]. The deterioration of cognitive functions, behavioral disturbances, and inadequate nursing and parasanitary training contribute to poor oral health in elderly people with dementia [18,19,20]. Studies show that frail people having fewer teeth are associated with a lack of nutritional supply and that dental status could play a role in musculoskeletal frailty [6,21] and cognitive impairment [22]; additionally, the presence of lesions of the oral mucosa could interfere with the nutrition of elderly patients [6,23,24,25]. The first alarm bell is the lack of energy that the elderly manifest during the day and that social health workers notice and report to the doctor in order to act preventively/promptly on potentially reversible causes [26,27]. In residences for the elderly, all food is controlled and administered to the residents according to a diet recommended by a nutritionist with geriatricians; not all residents are able to chew substantial food, and those who have few or no teeth find it difficult to crush food, even with their tongue. Re-establishing proper chewing with prosthetic rehabilitation would allow the frail elderly to be able to reactivate all the anatomical structures that would allow them to crush, grind and swallow food, even with the help of their tongue. Even if the force applied by natural teeth is greater than that applied by the prosthetic teeth of a mobile prosthesis, elderly people who can chew tend to prefer more consistent foods, unlike those who have few or no teeth, who tend to prefer soft foods, smoothies or, otherwise, sweet foods. Even the psychological aspect of the elderly who were heavily tested during the pandemic [28,29] varies according to the dental status; often, the elderly tend to no longer take care of aesthetic aspects, due to advanced age. The objective of this study was to perform the dental prosthetic rehabilitation of fragile subjects and to evaluate two aspects through the compilation of informative questionnaires: The first concerns both the improvement or difficulty during the feeding phase and possible conversation during meals; the second concerns the way in which the patient’s psychological aspect has changed during socialization with other residents.

## 2. Materials and Methods

**Figure 1:**
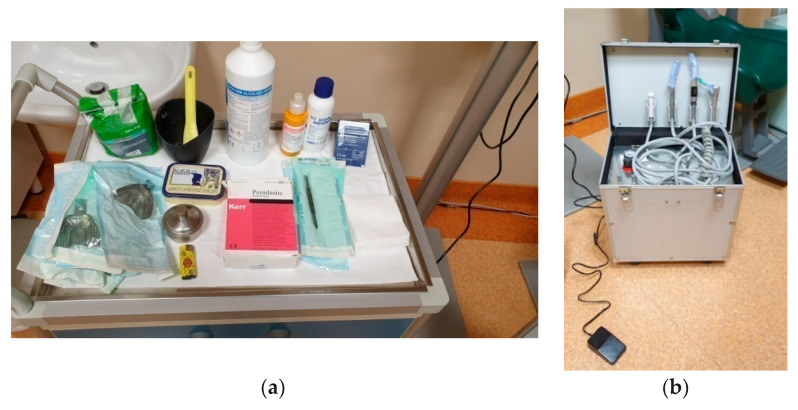
(**a**) Materials used for the study and relining impressions; (**b**) Portable unit complete with turbine, contra-angle, air-water syringe and saliva aspirator.

**Figure 2:**
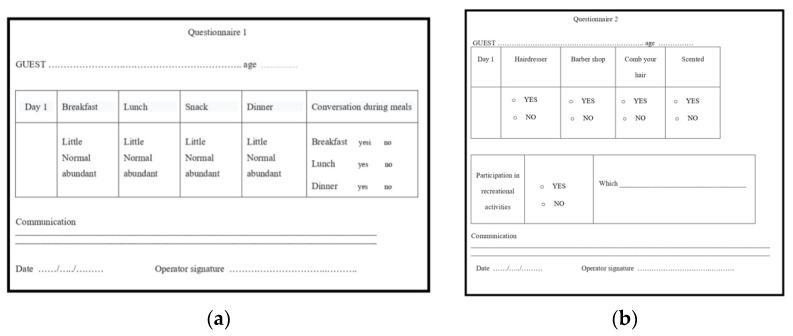
(**a**) Dossier 1; (**b**) Dossier 2.

**Figure 3:**
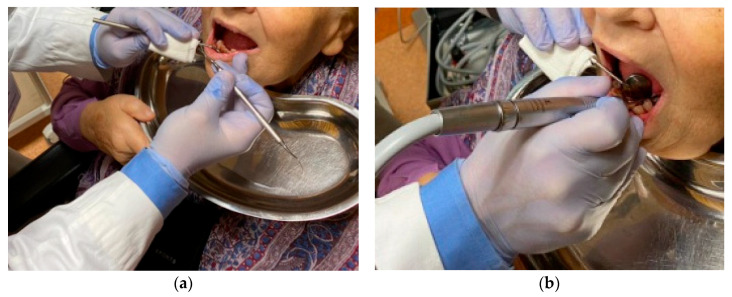
(**a**) Gingival probing; (**b**) Working phase with use of portable unit tool.

**Figure 4:**
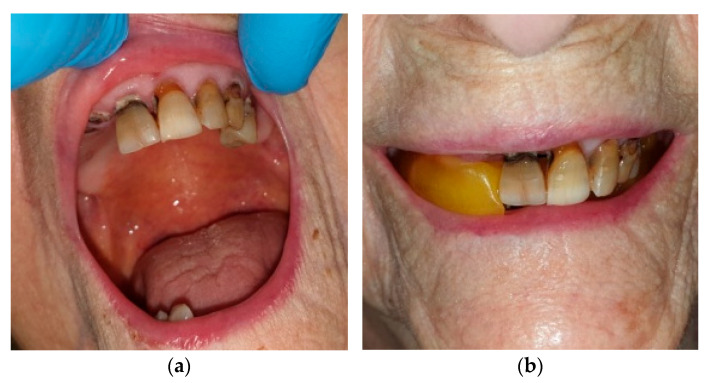
(**a**) Initial situation in a 99-year-old patient; (**b**) vertical-dimension registration phase.

**Figure 5:**
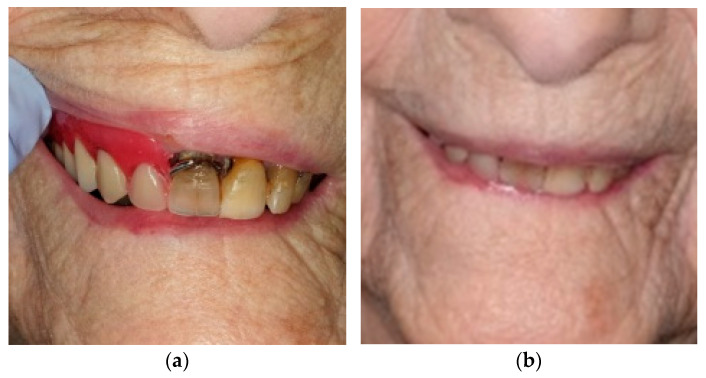
(**a**) Test of the removable prosthesis with teeth mounted on wax for any modifications; (**b**) Finished prosthesis.

At the request of the nursing home with dental interventions, a total of 75 patients were examined, and the research was conducted on only 67 patients, 26 men and 41 women, aged between 75 and 99, who were guests of 10 healthcare facilities (nursing homes) in the Lombardy district; only 8 residents have not undergone prosthetic rehabilitation, as they are not collaborating. All patients who were visited at the various nursing homes have expressed their written personal consent to the dental visit and to the prosthetic rehabilitation or modification of pre-existing prostheses (relining or repair), or have had consent expressed through their guardian. All patients, guardians or family members were informed of the completion of two information dossiers for the research by social health professionals and gave their consent. Patients were chosen for spontaneous and personal adherence to the visit and subsequent rehabilitation with prior written consent. All patients who requested a dentist were examined at the nursing homes’ residential facilities, and thus, the removable or removable prostheses were also made without having the patient move from the nursing homes. The patients were directly accompanied by the health and social workers to the room dedicated to dental operations; in addition, the health and social workers assisted the dentist during all the prosthetic rehabilitation phases. Dental interventions on residents were limited exclusively to solving prosthetic problems, and problems of an endodontic or surgical conservative nature were not supported by written consent; in any case, a dental facility or a room equipped to be a dental office would be needed. The residents we visited did not require surgery or endodontics, and the conservative operations were only performed after having been approved by the written consent of the relative-guardian or guardian. The materials and equipment used to make the removable prostheses were: alginate, wax, acrylic resin, composite resin, resin teeth, chromium-cobalt-molybdenum, a ruler, a marker, an articulator, a laboratory micromotor and a portable dental unit (Figure 1). The two information questionnaires, filled out by health and social workers, concerned the resident’s assessments during feeding (little power supply, normal feeding, abundant nutrition, conversation and during meals) and during play activities (Figure 2). The second information questionnaire also presents some aesthetic aspects relating to how the resident presented himself in order to engage in play activities (if women first went to the hairdresser, if men shaved their beard at the barber, if they asked for perfume, if men appeared combed). Having established the appointments for the prosthetic’s realization, we initially proceeded with the evaluation of the dental mobility of the residual teeth, the caries and the periodontium, eliminating plaque and tartar (Figure 3), and subsequently with the construction of the mobile prosthesis and with the removable or repair of the existing prostheses (Figure 4 and Figure 5). Any mobile elements or elements with destructive caries would have prevented the prosthetic realization, leading to a consequent variation of the treatment plan. All the prosthetic devices made were delivered in the morning by 11.30 to allow users to be able to use them immediately during the main meal. The two questionnaires, easily readable and understandable by the health and social staff, were completed after the main meals and after the play activities for a period of 30 days following the delivery of the prostheses. Before compiling the dossiers, all health and social workers reported that more than 50% of the selected patients had a normal social life within the nursing homes and that only a few actively participated in recreational activities.

## 3. Results

The patients who had not worn dental prostheses for at least two years numbered only 5 (3 men and 2 women), and the other 62 were wearing very old prostheses, with missing or worn teeth, broken hooks, or prostheses no longer adequate for adhesion and occlusion. The new made or repaired prosthetic devices have been met with great appreciation by the residents, who have been motivated by the fact that they have been able to use them immediately during the main meal at lunch. The made or repaired prosthetic devices are shown in Table 1. The results of the dossiers are shown in Table 2 and Table 3.

The compilation of the first dossiers highlighted how all the residents, despite the chewing difficulties with the new prosthesis, were fed and did not express any refusal to eat more consistent foods. Only 19.3% of men and 22% of women continued to eat little; moreover, during meals 35 residents conversed with other residents. The completion of the second dossier highlighted how the elderly who were frail had an extra incentive to improve the aesthetic aspect during socialization; furthermore, there was an improvement in participation in social activities among the residents, with a percentage of 73% of men and 88% of women.

## 4. Discussion

The difficulty for frail, non-self-sufficient or collaborating non-ambulatory patients residing in nursing homes to eat due to a lack of teeth or the absence of a dental prosthesis leads to an increasingly evident increase in patients’ systemic and psychological pathological state. In older individuals, reductions in the muscle mass of the geniohyoid, pterygoid, masseter, tongue and pharyngeal muscles have been documented. Swallowing is also a complex process, which involves numerous muscles of the face and neck in a coordinated manner; many changes related to aging, such as tissue elasticity, changes in the anatomy of the head, and reduced oral and pharyngeal sensitivity, can worsen swallowing, leading to so-called presbyophagy [30,31]. Older adults who experience dental problems often avoid solid foods such as meat, fruit and vegetables, which are major sources of protein, fiber, vitamins and minerals; in addition to the problems of malnutrition that these deficiencies entail, the soft foods selected by the elderly are foods rich in sugars and fats, which can further worsen chronic inflammation, cardiovascular risk and the onset of metabolic syndrome. A good interaction of the frail elderly with social health workers facilitates a recognition of the type of difficulty that the subject manifests during normal nutrition. Training social and healthcare workers from nursing homes on dental issues would strongly help the establishment’s medical staff, as they could intercept pathologies or lesions of the oral mucosa that bother the frail elderly and that thereby trigger a series of behaviors aimed at a reduced nutrition, resulting in malnutrition. In an evolving modern healthcare system where the digitization and application of new communication technologies has accelerated the ability to better respond (breaking down time barriers) to a medical need or immediate patient evaluation, it would be desirable in the near future to introduce telemedicine and videoconferencing for the elderly in nursing homes [32,33,34]. This tool would allow a rapid and constant evaluation of the possible prosthetic dental problems of elderly patients in social welfare residences [35,36,37].

## 5. Conclusions

Over time, the life lived by frail elderly patients in nursing homes forms a strong bond with those who are the last to see them constantly and talk with them during the day. Often, many frail elderly collaborators develop the art of getting by on their own, thus demonstrating to health and social workers an ability to still be able to solve problems. Several systemic factors contribute to poor oral health in the elderly, which, added to the lack of dental care, determine the state of malnutrition of the frail elderly. After implementing the prosthesis or after the repair of an existing prosthesis, each patient we treated showed different positive behaviors in normal daily life within their establishment, both in terms of food (in fact, 78% of women and 61.6% of men continued to eat without any problem) and in terms of an improvement in psychological balance; furthermore, during the visits, the relatives of the residents were able to notice a more positive attitude of their family members. This shows that frail elderly residents in healthcare facilities are subjects who also need to be followed dentistically; it also demonstrates that the frail elderly, even if they are not self-sufficient, have the opportunity to live the last years of their life sharing moments of socialization and fun with other seniors, if they are supported well. In order to guarantee complete assistance to these patients, residential care institutions should include specific dental protocols in their healthcare program.

## Figures and Tables

**Table 1:** Number and types of made or repaired prosthetic devices.

	1 Arch Mobile Prosthesis	2 Arch Mobile Prosthesis	1 Arch Skeletonized Prosthesis	2 Arch Skeletonized Prosthesis	Relining Repair
Men	8	2	4	3	9
Women	11	3	7	5	15

**Table 2:** Results of dossier n° 1.

	Little Power Supply	Normal Feeding	Abundant Nutrition	Conversation
N° Men	5	16	5	12 out of 26
N° Women	9	30	2	23 out of 41

**Table 3:** Results of dossier n° 2.

	Hairdresser	Barber Shop	Combing Hair	Scented
N° Men	--	26	16	20
N° Women	41	--	41	37
